# Multiple Comprehensive Analyses Identify the Protective Role and Diagnostic Signature of Mannose Metabolism in Ulcerative Colitis

**DOI:** 10.3390/ijms26199443

**Published:** 2025-09-26

**Authors:** Yunze Liu, Huizhong Jiang, Yixiao Gu, Yuan Li, Xia Ding

**Affiliations:** 1 The First Clinical Medical College, Beijing University of Chinese Medicine, Beijing 100029, China; 2 The First Clinical Medical College, Xuzhou Medical University, Xuzhou 221000, China; 3 School of Traditional Chinese Medicine, Beijing University of Chinese Medicine, Beijing 100029, China; 4 National Institute of TCM Constitution and Preventive Medicine, Beijing University of Chinese Medicine, Beijing 100029, China

**Keywords:** ulcerative colitis, mannose metabolism, immune cell, Mendelian randomization, machine learning

## Abstract

Metabolic reprogramming has recently been recognized as related to immune disorders in ulcerative colitis (UC), but the specific metabolic pathways and genes involved remain unclear. Here, Mendelian randomization confirmed that mannose and mannonate exhibited a negative causal relationship with UC, and that the immune cell phenotype HLA DR on CD33dim HLA DR+ CD11b− mediated the effect of mannonate on UC. Bulk RNA sequencing data revealed that mannose metabolism abnormity is critical for driving the innate and acquired immune response. A well-performing diagnostic model related to mannose metabolism was constructed using SVM analysis, achieving an AUC-ROC value of 0.987 in the training set and an AUC-ROC value of 0.899 in the validation set. Single-cell analysis revealed that epithelial cells in which the mannose metabolism pathway was inactivated demonstrated increased intercell communication with myeloid cells, T cells, and B cells. In vitro experiments confirmed that KHK and AKR1B10 were suppressed under inflammatory stimulation, which may hinder mannose-related metabolism. This study elucidates the protective role of mannose metabolism in UC and provides a novel gene signature for diagnosis and treatment.

## 1. Introduction

Ulcerative colitis (UC), a nonspecific inflammatory bowel disease with an unknown etiology, contributes to the risk of colon cancer. In the last few decades, the incidence of UC has been continuously increasing worldwide, especially in developed countries [1]. In North America, approximately 400 out of every 100,000 individuals are diagnosed with UC [2], which constitutes a major health concern. Despite the availability of commonly used drugs (such as mesalazine, sulfasalazine, etc.), UC continues to progress in many patients [3]. Elucidating the underlying mechanisms mediating the progression of UC is critical for disease control.

Ulcerative colitis is a condition characterized by dysfunctions within both the innate and adaptive immune systems [4,5]. Myeloid cells play a crucial role in initiating innate immune responses to pathogenic substances via pattern recognition receptors [6]. Myeloid cells can also affect adaptive immunity through the regulation of T cells. By serving as key antigen-presenting cells, myeloid cells increase T-cell activation, differentiation, and proliferation by increasing the presence of MHC molecules and providing the necessary costimulatory signals [7]. Dysregulation of intestinal myeloid cells in UC is closely linked to the onset and progression of this disease [8].

Current studies have shown that alterations in metabolite levels play critical roles in regulating the immune response and tissue homeostasis [9,10]. On the one hand, some metabolites, such as mannose, in healthy tissue can exhibit anti-inflammatory properties [11]. Mannose is a key component in the synthesis of glycoproteins and glycolipids and plays a crucial role in cell recognition and immune regulation. High levels of mannose can suppress inflammatory responses by limiting macrophage activation and affecting T-cell differentiation, whereas decreased levels of mannose contribute to inflammatory effects [12,13]. On the other hand, abnormal metabolic processes produce an abundance of harmful metabolites, such as reactive oxygen species (ROS), which negatively influence immune regulation and participate in UC progression [14].

**Figure 1:**
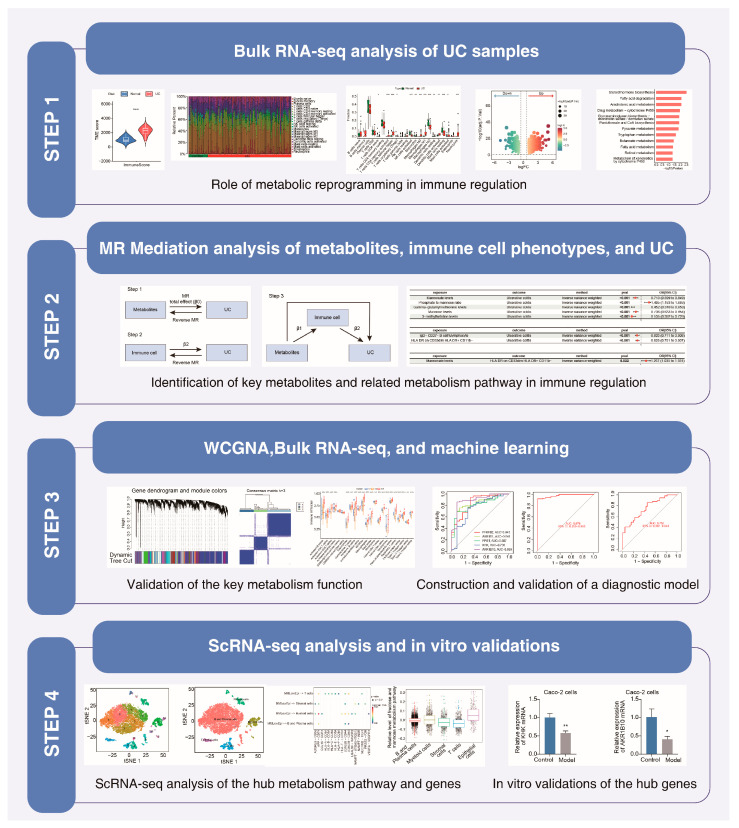
The study flowchart. * *p* < 0.05; ** *p* < 0.01.

Although observational and experimental studies have shown that metabolism dysregulation is a key driver of immune dysregulation, establishing definitive causal associations among metabolism, the immune response, and the pathological processes of UC remains challenging due to methodological limitations. In this study, we revealed marked immune dysregulation and metabolic disorders using transcriptomic data from UC patients. Importantly, we also conducted Mendelian randomization (MR) analysis to draw causal connections among metabolites, immune phenotypes, and UC risk. Furthermore, using the metabolites and metabolism genes found to be involved in immune regulation and UC progression, we constructed a machine learning model that showed significant value in disease diagnosis. Single-cell analysis further revealed the relationship between metabolic gene expression and the immune response. Finally, the experimental results verified the expression of key metabolism genes (Figure 1). This study aimed to explore the key metabolic–immune regulatory changes in UC, contributing to the elucidation of disease mechanisms and the development of novel diagnostic and therapeutic measures.

## 2. Results

### 2.1. Immune Abnormalities and Metabolic Reprogramming Involved in the Pathological Processes of UC

**Figure 2:**
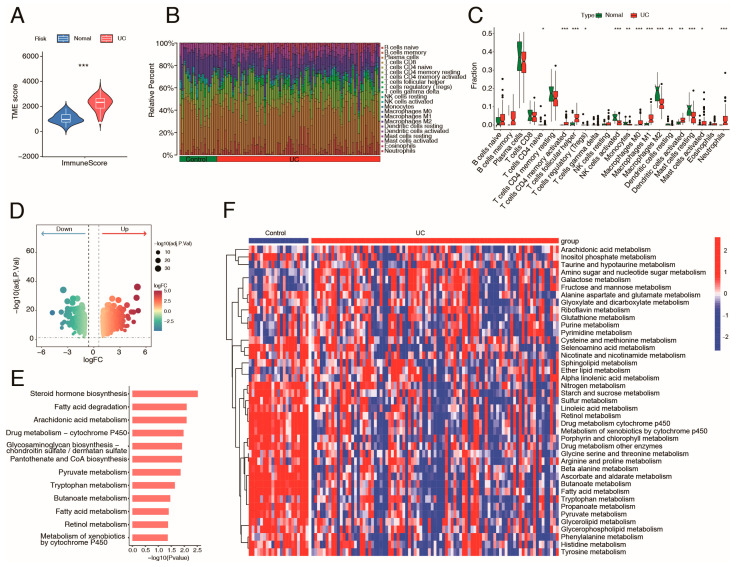
Immune abnormalities and metabolic programming involved in the pathological processes of UC. (**A**) Immune score of UC and normal samples (GSE87466). (**B**) Bar plot showing the proportion of infiltrating immune cells in the UC and normal control groups. (**C**) Boxplot concerning the differences in abundance of immune cells between the groups. (**D**) Volcano plot of DEGs between the UC and normal control groups. (**E**) KEGG analysis of the UC-related DEGs. (**F**) Heatmap of metabolic pathways between the groups. *, *p* < 0.05; **, *p* < 0.01; ***, *p* < 0.001.

Using the ESTIMATE algorithm, we calculated the immune score of UC samples (from the GSE87466 dataset), and the results revealed that patients with UC had significantly greater immune scores compared with the normal population (Figure 2A). Then, a thorough evaluation of the variations in immune cell abundances between patients with UC and normal intestinal tissues was conducted utilizing the CIBERSORT algorithm (Figure 2B,C). The findings depicted in Figure 2C indicated that immune cells, including neutrophils, M1 macrophages, and activated NK cells, were more abundant in patients with UC than in the normal population. In total, 1176 differentially expressed genes (DEGs) between UC and normal samples were identified (*p* < 0.05, |fold change (FC)| ≥ 1.5) (Figure 2D) and subsequently subjected to KEGG pathway analysis. As shown in Figure 2E, KEGG analysis revealed enrichment of various metabolic pathways, such as fatty acid degradation, arachidonic acid metabolism, and tryptophan metabolism. Furthermore, Gene Set Variation Analysis (GSVA) heatmap also revealed that various metabolic pathways were altered in the UC group (Figure 2F). These results suggest widespread dysregulation of metabolism and immunity in patients with UC.

### 2.2. Two-Sample MR Analysis of Metabolites and UC

**Figure 3:**
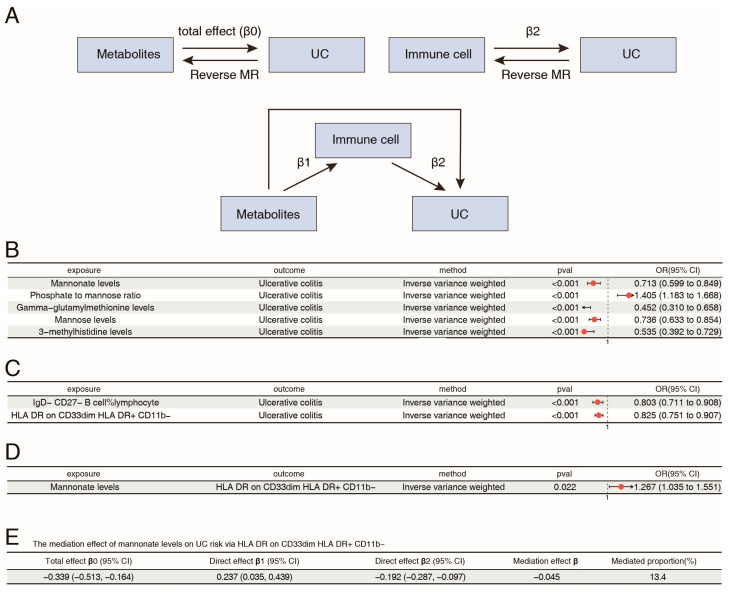
Mendelian randomization of associations between metabolites, immune cell phenotypes, and UC. (**A**) The flowchart of Mendelian randomization mediation study. (**B**) Forest plot of the causal relationship between metabolites and UC risk. (**C**) Forest plot of the causal relationship between immune cell phenotypes and UC risk. (**D**) Forest plot of the causal relationship between identified metabolites and immune cell phenotypes. (**E**) The mediation effect of mannonate levels on UC risk via HLA DR on CD33dim HLA DR+ CD11b−.

To further clear the key metabolic signatures linked to UC pathogenesis, the causal relationships between 1400 metabolites and UC using two-sample MR were evaluated (Figure 3A). After eliminating genes in linkage disequilibrium and weak IVs, we conducted inverse-variance weighted (IVW) analysis on the SNPs associated with plasma metabolites and applied FDR correction to the IVW results. Five plasma metabolite biomarkers, including mannonate levels, the phosphate-to-mannose ratio, gamma-glutamylmethionine levels, mannose levels, and 3-methylhistidine levels, were preliminarily identified as causally related to UC (Figure 3B). A reverse MR analysis was subsequently conducted utilizing the IVW and MR Egger methods, with UC as the exposure factor and the five metabolites as the outcome factors. As a result, UC did not have a reverse causal relationship with four of the five metabolites (mannonate levels, the phosphate-to-mannose ratio, 3-methylhistidine levels, and mannose levels) (*p* > 0.05) (Figure S1). Among the four metabolites, mannonate levels, 3-methylhistidine levels, and mannose levels were negatively associated with UC, whereas the phosphate-to-mannose ratio was positively correlated with UC (Figure 3B and Figure S2A–D). As shown in Table S1, the total causal effect of metabolite levels affecting UC was calculated (β0). Furthermore, leave-one-out sensitivity analysis and horizontal pleiotropy and heterogeneity tests confirmed the robustness of the MR findings (Figure S2E–H, Tables S2 and S3). The same causal trends were revealed by four additional supplementary methods (Figure S2I,L).

### 2.3. Mediation Analysis of Metabolites, Immune Cell Phenotypes, and UC

To explore whether the specific alterations in metabolism contributed to UC risk through affecting immune cell phenotypes, mediation MR analysis between metabolites, immune cell phenotypes, and UC was next conducted. According to the foundational principles of the mediation MR analysis, we calculated the effects of immune cell phenotypes on UC (β2). After eliminating genes in linkage disequilibrium and weak IVs, we conducted IVW analysis on the SNPs and applied FDR correction to the IVW results. Two immune cell phenotypes were correlated with UC (FDR < 0.05). IgD− CD27− %lymphocyte and HLA DR on CD33dim HLA DR+ CD11b− were negatively correlated with UC (Figure 3C and Figure S3A,B, Table S4). The same causal trends were revealed by four additional supplementary methods (Figure S3C,D). The results of leave-one-out sensitivity confirmed the reliability of these findings (Figure S3E,F). In addition, there was no significant horizontal pleiotropy or heterogeneity (*p* > 0.05) (Tables S5 and S6). A reverse MR analysis was subsequently performed, with UC as the exposure factor and the two immune cell phenotypes as the outcome factors. The results showed that UC did not have a reverse causal relationship with the two immune cell phenotypes (*p* > 0.05) (Figure S4).

Next, the effects of the above four metabolites on the two immune cell phenotypes were assessed using the IVW approach. One significant causal relationship was noted. Specifically, there was a positive correlation between mannonate levels and HLA DR on CD33dim HLA DR+ CD11b− (OR: 1.267 95% CI: 1.035 to 1.551), yielding an effect size β1 from the metabolite to the immune cell phenotype (Figure 3D and Figure S5A,B, Table S7). In addition, leave-one-out sensitivity analysis confirmed the reliability (Figure S5C). There was no significant horizontal pleiotropy or heterogeneity (Tables S8 and S9). Furthermore, the mediation effect was calculated. Specifically, mannonate affect UC through HLA DR on CD33dim HLA DR+ CD11b− cell phenotype, with mediated percentage of 13.4% (Figure 3E).

### 2.4. Identification of Key Metabolism Genes via WGCNA and GSVA

**Figure 4:**
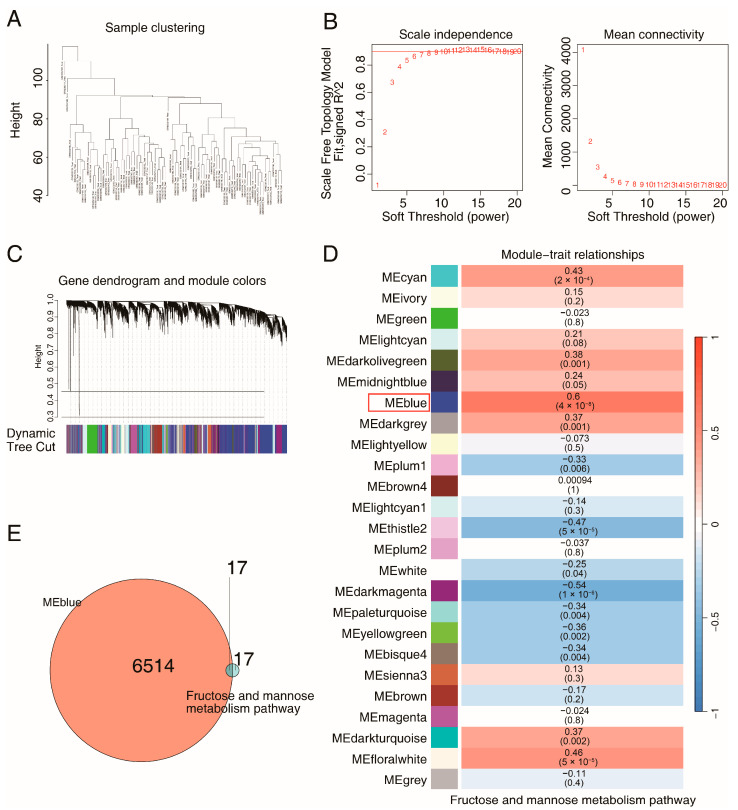
Identification of hub genes in UC using WGCNA. (**A**) Hierarchical clustering trees of mRNAs expression patterns in UC samples (GSE87466). (**B**) Topological calculations with soft thresholds value (1~20) to select the optimal soft threshold. (**C**) Clustering dendrograms of genes in UC. (**D**) Heatmap of the correlation analysis of module eigengenes with the fructose and mannose metabolism pathway(the MEblue module contains the genes that are the most strongly correlated with this pathway). (**E**) Venn diagrams for intersecting genes between coexpressed module genes and genes in the metabolism pathway.

To further identify mannonate-related metabolic genes, weighted gene coexpression network analysis (WGCNA) was conducted on colon samples from patients with UC (GSE87466), and WGCNA defines clusters of genes using an unbiased approach (Figure 4A). The topological calculation was performed by utilizing a soft threshold value that varied between 1 and 20. This analysis revealed that the optimal soft threshold value for the study was 6 (Figure 4B). Following this step, the gene expression matrix was transformed into an adjacency matrix. This adjacency matrix was then further converted into a topological overlap matrix (TOM). Next, average linkage hierarchical clustering was employed to classify the TOM-based modules, ensuring that each module contained a minimum of 60 genes. After the highly correlated modules were merged, a total of 25 modules were identified and named according to the module color displayed on the hierarchical clustering tree (Figure 4C). Furthermore, using Pearson analysis, we calculated the correlation effects between the gene modules and the fructose and mannose metabolism pathway (Figure 4D). The gene module with the greatest correlation effect was selected as the coexpressed gene module for the metabolism pathway. The mannonate-related genes were subsequently screened on the basis of the intersection of the coexpressed module genes and genes involved in the fructose and mannose metabolism pathway (Figure 4E), and 17 hub metabolism genes were ultimately identified.

### 2.5. Effects of Mannose Metabolism-Related Genes on Immune Infiltration in UC

**Figure 5:**
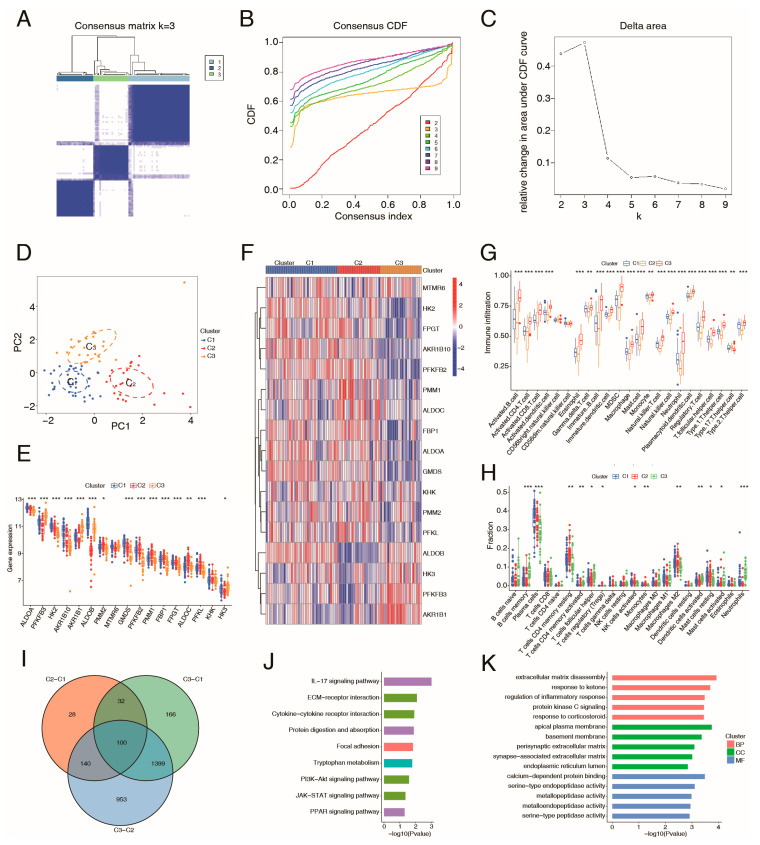
Consensus cluster analysis of the mannose metabolism-related genes in UC. (**A**) Consensus clustering matrix for UC (k = 3). (**B**) CDF plot from the consensus clustering analysis. (**C**) Delta area plot of consensus clustering. (**D**) PCA analysis showing the distribution of three gene clusters identified by consensus clustering. (**E**,**F**) Boxplot (**E**) and heatmap (**F**) of mannose metabolism-related gene expression in the three subgroups. (**G**) Boxplot of immuno-related function enrichment based on ssGSEA analysis. (**H**) Boxplot showing the proportion of infiltrating immune cells in the three subgroups. (**I**) Venn diagrams for intersecting differentially expressed genes across the three subgroups. (**J**) KEGG analysis of the differentially expressed genes. (**K**) GO analysis of the differentially expressed genes. *, *p* < 0.05; **, *p* < 0.01; ***, *p* < 0.001.

Next, a bioinformatics analysis was conducted to evaluate the effects of mannose metabolism on immune infiltration in UC. Based on the expression of the above 17 hub genes associated with mannose metabolism, we performed consensus clustering analysis to identify clusters of UC patients from the GSE87466 dataset. The UC cohort was categorized into three subgroups (Figure 5A–D). The heatmap and boxplots revealed significant differences in the expression of mannose metabolism-related genes among the three subgroups (Figure 5E,F). Single-sample gene set enrichment analysis (ssGSEA) was conducted to assess the variations in immune cell infiltration across different subgroups, demonstrating significant differences in the presence of macrophages, dendritic cells, neutrophil, T-cell subtypes, B-cell subtypes, and others (Figure 5G). Additionally, immune cell infiltration analysis using the CIBERSORT method indicated that the three subgroups exhibited markedly different immune infiltrates, including activated dendritic cells, M2 macrophages, and neutrophils (Figure 5H). Collectively, these findings supported the mendelian MR evidence suggesting that mannose metabolism could influence immune cell function and exert protective effects in UC.

To explore the mechanism underlying the distinct immune responses occur, we further analyzed the DEGs associated with various mannose metabolism patterns across the subgroups. The Venn diagram revealed 100 common DEGs among the three subgroups (Figure 5I), and the functions of these genes were subsequently assessed through KEGG and GO enrichment analyses. As shown in Figure 5J, KEGG analysis indicated that the DEGs are primarily enriched in pathways such as the lL-17 signaling pathway, ECM-receptor interaction, and cytokine-cytokine receptor interaction. The GO enrichment results for biological processes (BPs) analysis demonstrated that the DEGs are involved in extracellular matrix disassembly, response to ketone, and regulation of inflammatory response, among others. The GO enrichment results for cellular components (CCs) included the apical plasma membrane, basement membrane, and perisynaptic extracellular matrix, among others. The GO enrichment results for molecular functions (MFs) included calcium-dependent protein binding, serine-type endopeptidase activity, and metallopeptidase activity, among others (Figure 5K). These findings identified the downstream pathways through which mannose metabolism may exert its influence.

### 2.6. Construction and Verification of a Metabolism-Related Diagnostic Model for UC Patients via Machine Learning

**Figure 6:**
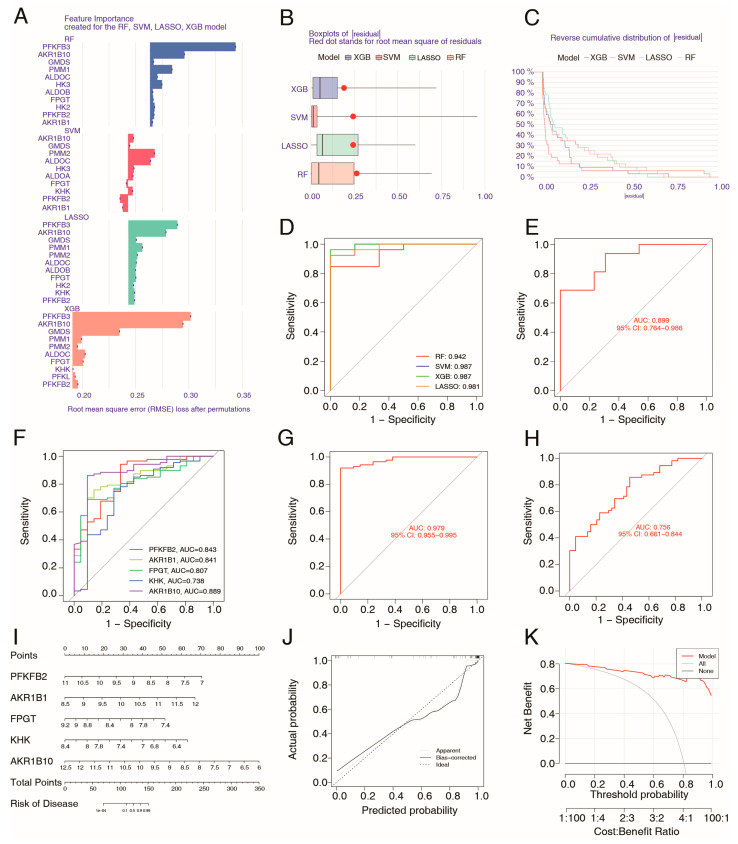
Construction and verification of a metabolism-related diagnostic model for UC patients via machine learning. (**A**) The importance of variables in each machine learning model. (**B**) Box plots of residual of each machine learning model. (**C**) Reverse cumulative distribution of residual showing the residual distribution. (**D**) ROC curves of these four models based on the 17 metabolism genes (training set). (**E**) ROC curves of the SVM model based on the 17 metabolism genes (validation set). (**F**) ROC curves of the SVM model based on each of the top five genes (training set). (**G**) ROC curves of SVM model based on the total of top five genes (training set). (**H**) ROC curves of SVM model using the top five genes (validation set). (**I**) A nomogram that predicts UC risk based on a five-gene SVM model. (**J**,**K**) Calibration (**J**) and DCA (**K**) curve of the nomogram model using the top five metabolism genes.

Given the critical role of mannose metabolism in the pathogenesis of UC, we utilized the 17 identified mannose metabolism-related genes to develop a novel diagnostic model for UC. Four types of machine learning classification algorithms (random forest [RF], XGBoost [XGB], support vector machine [SVM], and least absolute shrinkage and selection operator [LASSO]) were employed for model construction using the training set (GSE87466). We analyzed the relative importance of each feature gene in the four machine learning models using the DALEX method and sorted the significant genes by relative importance in descending order (Figure 6A). The classification models were built, and the residual analysis results suggested that the SVM model is the most suitable model (Figure 6B,C). The performance of each model was evaluated using receiver operating characteristic (ROC) curves along with the area under the ROC curve (AUC-ROC) as a quantitative measure. As shown in Figure 6D, RF analysis yielded an AUC-ROC score of 0.942, XGB analysis resulted in an AUC-ROC value of 0.987, SVM analysis yielded an AUC-ROC value of 0.987, and LASSO analysis yielded an AUC-ROC value of 0.981. The ROC curve results demonstrated that these metabolic genes had excellent diagnostic efficacy in UC patients, and the best-performing SVM model was selected for further validation analysis. The validation set (GSE36807) was used to confirm the diagnostic signature of the SVM model, which resulted in an AUC-ROC value of 0.899 (Figure 6E).

Next, the top five metabolism genes (PFKFB2, AKR1B1, FPGT, KHK, and AKR1B10) with the best performance were screened out by the SVM algorithm (Figure 6F). A SVM model was reconstructed based on the five genes, which also displayed good prediction performance in both the training set and the validation set (Figure 6G,H). A nomogram was established for predicting UC risk based on the sum of each gene point (Figure 6I). Calibration plot and decision curve analysis (DCA) confirmed the prediction efficiency of the nomogram (Figure 6J,K). These results revealed that variations in the expression of these genes contribute to the pathogenesis of UC and that these expression patterns can serve as potential biomarkers for UC risk stratification.

### 2.7. ScRNA-Seq Analysis of Metabolism–Immune Relationships in UC Samples

**Figure 7:**
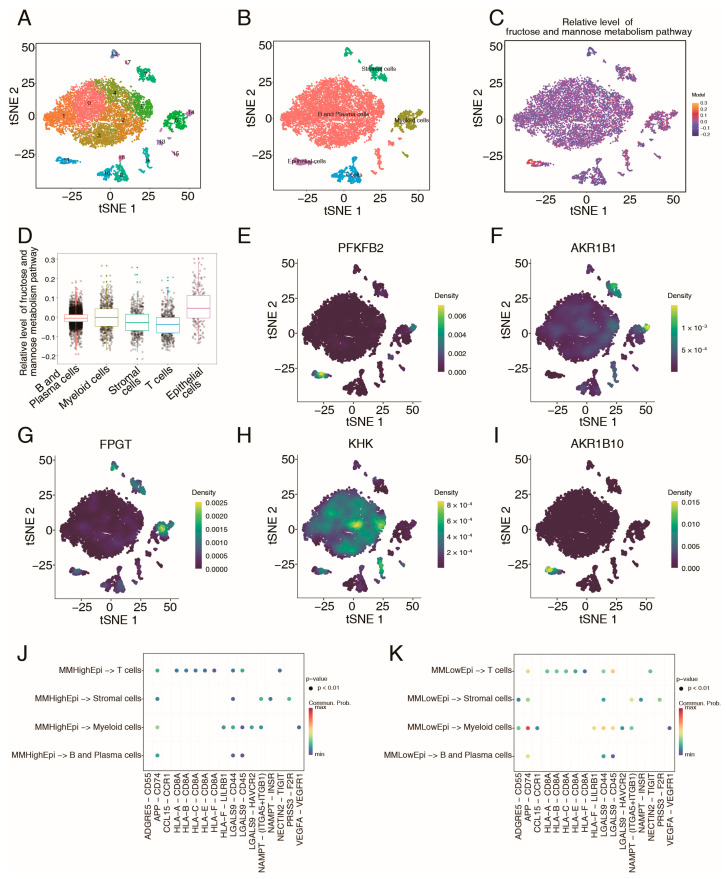
ScRNA-seq analysis of the metabolism genes. (**A**) t-SNE plot displaying 17 subgroups after dimension reduction (GSE214695). (**B**) t-SNE plot showing annotated cell categories. (**C**,**D**) t-SNE plot (**C**) and box plot (**D**) displaying the activation level of the fructose and mannose metabolism pathway in various types of cells. (**E**–**I**) t-SNE plots showing the distribution of the metabolism genes. (**J**) Single-cell ligand-receptor analysis showing intercell communications between epithelial cells with activation of fructose and mannose metabolism pathway (MM High Epi) and other cells. (**K**) Single-cell ligand-receptor analysis showing intercell communications between epithelial cells with inactivation of fructose and mannose metabolism pathway (MM Low Epi) and other cells.

To further understand the role of mannose metabolism and the hub genes in UC, a single-cell UC atlas (GSE214695) was constructed. After the quality control procedures were completed and the data were merged, a total of 17,969 cells were analyzed utilizing the Seurat algorithm. PCA and t-SNE analysis were then performed for dimension reduction, and the cells were clustered into 17 distinct subgroups (Figure 7A). Cell type annotation was further conducted using the “Single R” method and previously published studies, which divided 17 subgroups into five cell types, namely, stromal cells, epithelial cells, T cells, B cells, plasma cells, and myeloid cells (Figure 7B). We investigated the activation levels of the fructose and mannose metabolism pathways in various types of cells. As shown in Figure 7C,D, this metabolic pathway was highly activated in intestinal epithelial cells. Additionally, the single-cell atlas revealed that most of the hub metabolism genes were highly expressed in epithelial cells (Figure 7E–I).

**Figure 8:**
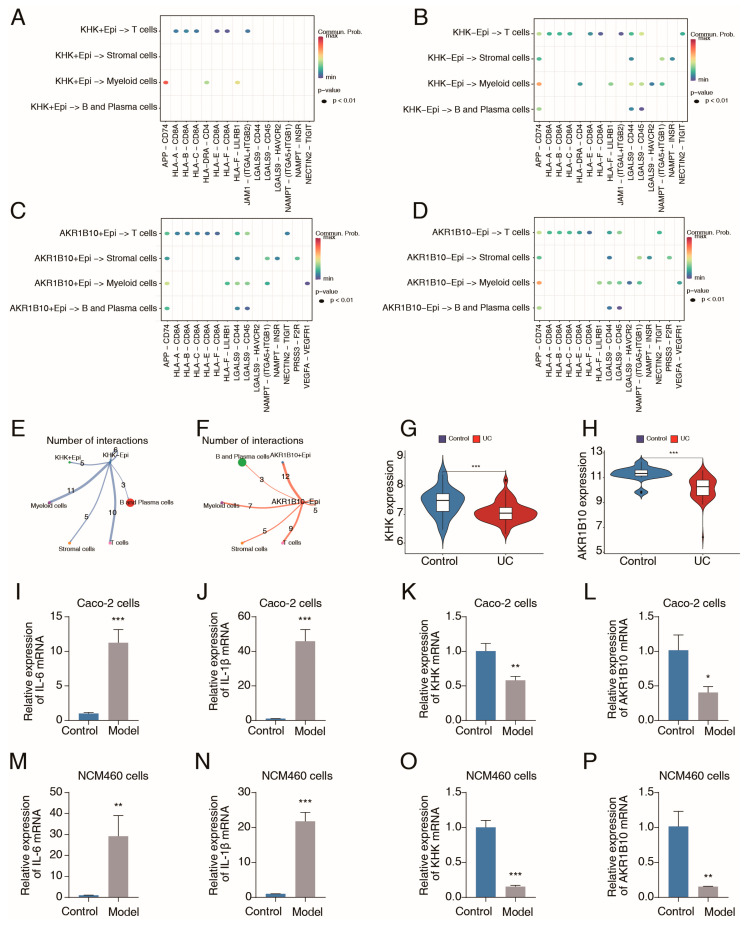
scRNA-seq analysis of the gene function and validation of metabolism gene expression in inflammatory colon epithelial cells. (**A**,**B**) Single-cell ligand-receptor analysis showing intercell communications between epithelial cells with high or low expression of KHK (KHK+ or KHK− Epi) and other cells. (**C**,**D**) Single-cell ligand-receptor analysis showing intercell communications between epithelial cells with high or low expression of AKR1B10 (AKR1B10+ or AKR1B10− Epi) and other cells. (**E**,**F**) Global intercellular communication among subsets based on number of interactions. (**G**,**H**) Violin plots of KHK (**G**) and AKR1B10 (**H**) expression in normal colon samples and UC patient tissues in the GSE87466 dataset. (**I**,**J**) qRT-PCR analysis of IL-6 (**A**) and IL-1β (**B**) mRNA expression in normal Caco-2 cells and Caco-2 cells following the TNF-α stimulation (model) (n = 3). (**K**,**L**) qRT-PCR analysis of KHK (**C**) and AKR1B10 (**D**) mRNA expression in normal and model Caco-2 cells (n = 3). (**M**,**N**) qRT-PCR analysis of IL-6 (**E**) and IL-1β (**F**) mRNA expression in normal NCM460 cells and NCM460 cells following the TNF-α stimulation (model) (n = 3). (**O**,**P**) qRT-PCR analysis of KHK (**G**) and AKR1B10 (**H**) mRNA expression in normal and model NCM460 cells (n = 3). Data are shown as the mean ± SD (*, *p* < 0.05; **, *p* < 0.01; ***, *p* < 0.001).

Next, the role of the fructose and mannose metabolism pathway in epithelial cells in immunoregulation was investigated through single-cell ligand–receptor analysis. Interestingly, various inflammatory ligand–receptor signals, including the APP−CD74 axis, LGALS9−CD45 axis, and LGALS9−CD44 axis, were predominantly activated between epithelial cells with inactivation of the fructose and mannose metabolism pathway and myeloid cells, whereas these signals were relatively inactivated between epithelial cells with activation of the fructose and mannose metabolism pathway and myeloid cells (Figure 7J,K). Interactions between epithelial cells and other immune cells (T cells, B cells, and plasma cells) were also enhanced when the fructose and mannose metabolism pathway was inactivated (Figure 7J,K). Additionally, we explored the roles of the hub genes KHK and AKR1B10 in immunoregulation. Epithelial cells exhibiting low expression levels of KHK or AKR1B10 generally presented increased inflammatory ligand–receptor signals (Figure 8A–D). The results of cell communication analyses indicated a close relationship between immune cells and epithelial cells with reduced expression of KHK or AKR1B10 (Figure 8E,F). These findings suggested that the fructose and mannose metabolism pathway in epithelial cells may confer protection against inflammatory damage by limiting intercellular communication between epithelial and immune cells, which aligns with our MR results.

### 2.8. Validation of Metabolic Gene Expression in UC Samples and Inflammatory Colon Epithelial Cells

The expression levels of KHK and AKR1B10 were evaluated in normal colon samples and tissues from patients with UC using the GSE87466 dataset. Both KHK and AKR1B10 exhibited relatively low expression in the UC patient tissues (Figure 8G,H). Given that scRNA-seq revealed that the hub metabolism genes were expressed in epithelial cells, the expression level differences in these genes between normal and inflammatory colon epithelial cells were further investigated, with commonly used UC epithelial cell models (the TNF-α–induced Caco-2 cell model and the TNF-α–induced NCM460 cell model). The qRT–PCR results revealed that the IL-6 and IL-1β levels were significantly elevated in Caco-2 cells following TNF-α stimulation, confirming the successful establishment of the cell model (Figure 8I,J). KHK and AKR1B10 mRNA levels were significantly decreased in inflammatory Caco-2 cells (Figure 8K,L). Similarly, the levels of IL-6 and IL-1β were significantly upregulated, while KHK and AKR1B10 expression was notably downregulated in TNF-α–induced NCM460 cells (Figure 8M–P). These findings indicated that KHK and AKR1B10 were suppressed under inflammatory stimulation, which may hinder mannose-related metabolism (Figure S6).

## 3. Discussion

An increasing number of studies have shown that metabolic reprogramming is related to immune disorders and inflammatory diseases. However, it remains unclear which specific metabolic processes are involved in immune abnormalities and UC risk and whether these associations are causal. To explore these questions, we employed an integrated application of bioinformatics based on multiomics data, MR analysis, and machine learning methods. To our knowledge, this study is the first to establish causal associations of metabolism-immunity regulation with the pathogenesis of UC through MR analysis. We also identified key metabolism genes and developed a novel metabolism-related diagnostic signature with promising clinical application in UC patients.

Blood metabolites are ideal for identifying biomarkers in UC screening because blood samples can be easily and repeatedly obtained. This study confirmed causal relationships between four plasma metabolite biomarkers and UC risk: mannonate, the phosphate-to-mannose ratio, 3-methylhistidine, and mannose. Notably, three of the four identified metabolic biomarkers were associated with mannose and the mannose metabolism pathway, and these findings revealed that mannose and the mannose metabolism pathway have a protective effect against UC. Mannose, a naturally occurring sugar, is commonly found in the diet [15]. Mannose metabolism is intricately associated with fructose metabolism through isomerization reactions, and mannose and fructose metabolism are highly coregulated [16]. Previous studies have reported that supplementation with mannose at safe supraphysiological doses can ameliorate inflammation and pathological lesions [17]. This study provides the first MR evidence that mannose has a protective effect on UC risk. In addition, our results revealed the negative impact of phosphate on UC. Phosphate is required for various biological activities, such as cell membrane construction, energy metabolism, and signal transduction. Some studies have reported that a disturbed phosphate balance can promote the development of metabolic syndrome and that long-term hyperphosphatemia is linked to inflammation [18].

Recent evidence has indicated that metabolic reprogramming is a key factor leading to immune disorders in patients with inflammatory diseases [19,20]. Cells rely on a multitude of nutrients and their metabolites to meet bioenergetic needs while maintaining necessary cellular functions. When inflammation occurs, various types of cells in the inflammatory microenvironment, such as immune cells and epithelial cells, always undergo metabolic reprogramming. Immune cells can acquire proinflammatory or anti-inflammatory phenotypes in response to different metabolic changes. One representative example is macrophages, which are capable of reprogramming metabolism pathways to produce proinflammatory M1 or anti-inflammatory M2 immune phenotypes [21]. In epithelial cells stimulated by inflammatory factors, metabolic reprogramming can induce inflammatory cascades, leading to further damage. For example, the levels of the metabolic byproducts ROS are significantly increased in UC patients [22]. Reactive oxygen species act as secondary messengers to cause cellular damage and even cell death via ROS-induced MAPK signaling [23,24]. In addition, abnormal metabolic patterns can severely affect signaling crosstalk between epithelial and immune cells, thereby contributing to immune disorders [25]. Together, metabolism and immunity form a complex regulatory network in UC patients, but current knowledge of this network is limited. Our study found that mannose-related metabolism can affect the myeloid cell phenotype CD33dim HLA DR+ CD11b−CD11b–, suggesting that the normal operation of mannose-related metabolism is crucial for maintaining immune homeostasis.

In the metabolism-related biomarker screening process, we adopted emerging techniques, including WGCNA and machine learning. Compared with conventional differential analysis, which identifies disease-associated genes according to their statistically significant differential expression, WGCNA is considered more conducive to discovering genes that act as hubs [26]. Here, WGCNA identifies highly correlated gene sets (modules) by constructing a gene coexpression network. By analyzing the relationships between these modules and metabolism pathway trait, we successfully identified key genes that play significant roles in regulating metabolism and are integral to disease-related biological networks. Accurate diagnosis can minimize medical resource waste, increase medical care efficiency, and reduce patient burden. Here, machine learning algorithms were used to identify final hub metabolism-related markers and construct a UC diagnosis model with stability and accurate predictive capabilities in both the training set and the external validation dataset.

This study revealed that the fructose and mannose metabolism pathway was activated in intestinal epithelial cells using single-cell data, and the hub genes were also highly expressed in epithelial cells. Furthermore, we found that this metabolism pathway played a key role in the cellular communication network between epithelial cells and immune cells. Various inflammatory ligand–receptor signals were relatively activated between epithelial cells with inactivation of the fructose and mannose metabolism pathway and myeloid cells. A decrease in the mannose metabolism-related genes KHK or AKR1B10 also contributes to the cellular communication network. A previous study revealed that AKR1B10 expression is significantly decreased in 77.3% of UC patient tissues [27]. AKR1B10 is a multifunctional aldo-keto reductase that is specifically expressed in colonic epithelial cells. AKR1B10 can activate GPX4, which can reduce the levels of various oxidative substrates (such as organic and hydrogen peroxides as well as hydroperoxides), thereby protecting cells from ROS-triggered cellular damage [28,29]. The inhibition of AKR1B10 expression leads to the accumulation of toxic ROS, thereby affecting the immune microenvironment [27,30]. KHK, as a rate-limiting enzyme for fructose, can regulate fructose metabolism, thereby influencing mannose metabolism [31]. Subsequent in vitro experiments confirmed that the expression of KHK and AKR1B10 was significantly downregulated in inflammatory intestinal epithelial cells. These results suggest that the maintenance of normal mannose metabolism is crucial for the immune balance of the local intestinal microenvironment. However, decreased expression of the mannose metabolism genes KHK and AKR1B10 mediated by inflammatory factor stimulation may suppress mannose metabolism, leading to immune disorders and UC risk. Thus, activating KHK and AKR1B10 may be a potential therapeutic strategy for UC.

In summary, this study elucidates key metabolic–immune regulatory mechanisms that play important roles in the pathogenesis of UC. These findings identify concrete biomarkers and therapeutic candidates at the metabolic level, which provides promising directions for future basic and clinical research.

## 4. Materials and Methods

### 4.1. Data Acquisition

RNA sequencing datasets GSE87466 (21 normal and 87 UC samples) and GSE36807 (7 normal and 15 UC samples), along with their corresponding clinical information, were retrieved from the GEO database (https://www.ncbi.nlm.nih.gov/geo/, accessed on 11 December 2024). Additionally, the single-cell RNA-seq dataset GSE214695 (6 UC samples) was also downloaded from GEO. The aforementioned datasets adhered to the access rules of GEO. The “c2.cp.kegg.v7.4.symbols” dataset was sourced from the MSigDB database (https://www.gsea-msigdb.org/gsea/msigdb, accessed on 11 December 2024) for metabolism-related pathways. The GWAS data for the 731 immune cell phenotypes and 1400 plasma metabolites utilized in this study were acquired from public repositories (GCST0001391 to GCST0002121 [3757 individuals] and GCST90199621 to GCST90201020 [8299 individuals], respectively) [32,33]. Furthermore, the GWAS data pertinent to UC was sourced from the IEU (ieu−a−973) (6687 UC and 19,718 normal samples).

### 4.2. Identification of the Immune Microenvironment and Consensus Clustering Analysis

R (University of Auckland, Auckland, New Zealand; version 4.3.2) were used for the analyses. The immune cell infiltration levels, referred to as the immune score, of UC samples were evaluated using the “ESTIMATE (version 1.0.13)” package [34]. The DEGs among immune-related genes were identified utilizing the “Limma (version 3.26.8)” package. Consensus clustering of the patients was performed using the “ConsensusClusterPlus (version 1.73.0)” package [35]. The proportions of infiltrating immune cells were determined using the CIBERSORT algorithm via the “CIBERSORT https://cibersortx.stanford.edu/ (accessed on 11 December 2024)” package [36].

### 4.3. Instrumental Variable Selection

The “TwoSample MR (version 0.5.6)” package was utilized to filter single nucleotide polymorphisms (SNPs) associated with the exposure, applying a significance threshold of *p* < 1 × 10^−5^ [37]. Subsequently, SNPs exhibiting linkage disequilibrium were excluded based on the criteria of R^2^ < 0.001 and a distance of 10,000 Kb. Additionally, SNPs with mismatched allele frequencies between the exposure and outcome, as well as palindromic SNPs with ambiguous strands, were removed, as previously described. Finally, SNPs with an F statistic greater than 10 were selected to mitigate the influence of weak instrumental variables (IVs) [38].

### 4.4. Two-Sample MR Analysis

Two-sample MR analysis was performed using the “MendelianRandomization” and “TwoSample MR” packages. The primary method employed for the MR analysis was the random-effects IVW method. The results indicated statistical significance when the p value of IVW was less than 0.05 and when the directions of the estimates obtained by the two methods (IVW and MR–Egger) were consistent. Risk was quantified using odds ratios (ORs) along with 95% confidence intervals (CIs). Additionally, other methods for two-sample MR, including MR–Egger, weighted mode, weighted median, and simple mode, were utilized for reference. Both the IVW and MR–Egger methods were used for reverse Mendelian randomization analysis, and the results indicated statistical significance when *p* < 0.05.

### 4.5. Mediation Analysis

Initially, two-sample MR analysis was employed to evaluate the causal effects (total effect, β0) of 1400 metabolites (exposure factors) on the risk of UC as the outcome. Subsequently, reverse MR analysis was conducted to identify metabolites that are non-causal. Following this, two-sample MR was utilized to assess the causal relationship between immune cells and UC (β2), as well as the effect of metabolites on immune cells (β1). This was followed by the calculation of the mediation effect (β1*β2), which represents the effect of each metabolite on UC risk through immune cells.

### 4.6. Sensitivity Analyses

The sensitivity analyses primarily included heterogeneity tests (Cochran Q test), assessments of horizontal pleiotropy (MR-Egger intercept method), and leave-one-out analysis. The leave-one-out analysis was conducted to determine whether the MR results were influenced or biased by potentially pleiotropic SNPs by sequentially removing each SNP.

### 4.7. Weighted Gene Coexpression Network Analysis

Hub metabolism genes in UC were identified using WGCNA with the gene matrix from the GSE87466 dataset [26]. The network topology was analyzed with a soft-threshold power (1~20) to determine the optimal soft threshold. The gene expression matrix was subsequently transformed into an adjacency matrix, which was then converted into a TOM. Average linkage hierarchical clustering was employed to classify the TOM-based modules, ensuring that each module contained a minimum of 60 genes. Similar modules were merged, and finally, the correlation between the combined modules and GSVA pathway results was calculated using the Pearson correlation method.

### 4.8. Establishment of Machine Learning Prediction Models

The RF, SVM, XGB, and LASSO models were developed using the “DALEX (version 2.4.3)” package, and ROC curves were used to evaluate the accuracy of these models. Additionally, the “rms (version 6.8-2)” and “ggDCA (version 4.1.3)” packages were utilized to construct the Nomogram plot, Calibration curve, and Decision Curve Analysis (DCA) curve for evaluating the diagnostic efficiency of the model.

### 4.9. ScRNA-Seq Analysis of Hub Metabolism Genes

The “Seurat (version 4.3.0)” package (https://satijalab.org/seurat/; accessed on 27 March 2023) was employed to analyze the scRNA-seq data (GSE214695) [39]. The scRNA-seq data were filtered based on the following criteria: less than 20% mitochondrial counts and more than 200 detected genes. The “harmony (version 1.2.3)” package was utilized for batch correction and subsequent data integration. Subsequently, the RunPCA and t-SNE methods were applied for dimensionality reduction and cell clustering. The top 20 principal components were selected, and a resolution of 0.8 was adopted for cluster identification. Clusters were annotated based on known marker genes from previously published studies and the “SingleR” method. Furthermore, the communication among these cell types was assessed using the “CellChat (version 2.1.0)” R package [40]. CellChat assessed the likelihood of communication at the level of signaling pathways by consolidating the communication probabilities from every ligand–receptor interaction linked to each specific signaling pathway.

### 4.10. Cell Culture

Caco-2 and NCM460 cells were obtained from the Cell Resource Center (National Infrastructure of Cell Line Resource, Beijing, China). For in vitro investigations, NCM460 cells were cultured in DMEM medium (Gibco, Carlsbad, CA, USA) supplemented with 10% fetal bovine serum (Gibco) and 1% penicillin/streptomycin (Gibco) at 37 °C in a 5% CO_2_ atmosphere. Caco-2 cells were cultured in DMEM (Gibco, Carlsbad, CA, USA) with 20% fetal bovine serum (Gibco) and 1% penicillin/streptomycin (Gibco) at 37 °C in a 5% CO_2_ atmosphere. Inflammatory Caco-2 and NCM460 cells were modeled using TNF-α. After the cells were fully grown, they were treated with 200 ng/mL TNF-α for 24 h to establish the model.

### 4.11. RNA Extraction and Quantitative Real-Time PCR (qRT-PCR) Assay

Total RNA samples from cells or tissues were extracted in accordance with the manufacturer’s instructions using the RNAeasy™ Animal RNA Isolation Kit with Spin Column (Beyotime, Shanghai, China). Subsequently, total cDNA was synthesized for quantitative PCR (qPCR) using the HiScript III 1st Strand cDNA Synthesis Kit (Vazyme, Nanjing, China). The quantitative real-time PCR assay was performed utilizing the Taq Pro Universal SYBR qPCR Master Mix (Vazyme, Nanjing, China). The expression levels of the target genes were measured by the 2^−ΔΔCt^ method, and GAPDH was used as the normalization control to ensure the accuracy of the quantification. To facilitate this analysis, all the primers used in the study were sourced from Sangon Biotech, which is located in Shanghai, China. A comprehensive list of these primers can be found in Supplementary File S1.

### 4.12. Statistical Analysis

All bioinformatics analyses were performed using R (version 4.3.0). For pairwise comparisons, normally distributed variables were analyzed using a nonpaired *t* test, whereas nonnormally distributed variables were analyzed via Wilcoxon’s nonparametric test. The statistical significance threshold was set at *p* < 0.05.

## 5. Conclusions

In this study, we identified mannose and mannose-related metabolites that exhibit a causal relationship with UC and confirmed that the immune cell phenotype characterized by HLA DR on CD33dim HLA DR+ CD11b− mediated the effect of mannonate on UC risk. Bioinformatics analysis of clinical UC samples revealed that abnormal mannose metabolism is critical for driving the response of immune cells. We further developed a novel diagnostic model related to mannose metabolism and identified five key genes (KHK, AKR1B10, etc.) involved in this metabolic pathway. Subsequent scRNA-seq analysis revealed that KHK and AKR1B10 in epithelial cells can limit cross-linking between epithelial cells and immune cells, which may explain why the mannose metabolism pathway prevents excessive nonspecific inflammation in UC. Further experimental results confirmed that KHK and AKR1B10 expression is inhibited under inflammatory stimulation, which may result in disordered mannose metabolism. In summary, our findings systematically elucidate the protective role of mannose metabolism in UC and provide a novel gene signature related to mannose metabolism for the diagnosis and treatment of UC.

## Data Availability

The original data presented in the study can be found in the online repositories (listed in the article). The data are also available from the corresponding author.

## Supplementary Materials

The following supporting information can be downloaded at: https://www.mdpi.com/article/10.3390/ijms26199443/s1.

## Abbreviations

AIC: Akaike information criterion; AUC: areas under the curve. AUC-ROC: area under the ROC curve; CI: confidence intervals; DCA: decision curve analysis; DEGs: differentially expressed genes; FDR: false discovery rate; GSEA: gene set enrichment analysis; GEO: gene expression omnibus; GSVA: gene set variation analysis; IVs: instrumental variables; IVW: inverse-variance weighted; KEGG: Kyoto Encyclopedia of Genes and Genomes; LASSO: least absolute shrinkage and selection operator; MSigDB: molecular signature database; MR: mendelian randomization; OR: odds ratios; PCA: principal component analysis; qRT–PCR: quantitative real-time polymerase chain reaction; RF: random forest; RNA-seq: RNA-sequence; ROC: receiver operator characteristic; scRNA-seq: single-cell RNA sequencing; ssGSEA: single-sample gene set enrichment analysis; SVM: support vector machine; TOM: topological overlap matrix; t-SNE: t-distributed stochastic neighbor embedding; UC: ulcerative colitis; WGCNA: weighted gene coexpression network analysis; XGB: XGBoost.
