# Supplementary material for: Multiple Comprehensive Analyses Identify the Protective Role and Diagnostic Signature of Mannose Metabolism in Ulcerative Colitis
Source: Int J Mol Sci. 2025 Sep 26;26(19):9443. doi: 10.3390/ijms26199443 (PMC12524464; doi:10.3390/ijms26199443)
Supplement: Supplementary file 1 [file ijms-26-09443-s001.zip › Supplementary figures.pdf]

| exposure | outcome | method | pval | OR(95% CI) |
|---|---|---|---|---|
| Ulcerative colitis | Gamma−glutamylmethionine levels | MR Egger | **0.030** | 1.057 (1.006 to 1.111) |
| Ulcerative colitis | Mannonate levels | Inverse variance weighted | 0.376 | 0.990 (0.967 to 1.013) |
| Ulcerative colitis | Mannose levels | Inverse variance weighted | 0.854 | 0.998 (0.974 to 1.022) |
| Ulcerative colitis | Phosphate to mannose ratio | Inverse variance weighted | 0.510 | 1.008 (0.984 to 1.032) |
| Ulcerative colitis | 3−methylhistidine levels | Inverse variance weighted | 0.287 | 0.987 (0.964 to 1.011) |

**Figure S1.** Forest plot showing the reverse Mendelian randomization analysis of the causal relationship between metabolites and UC risk

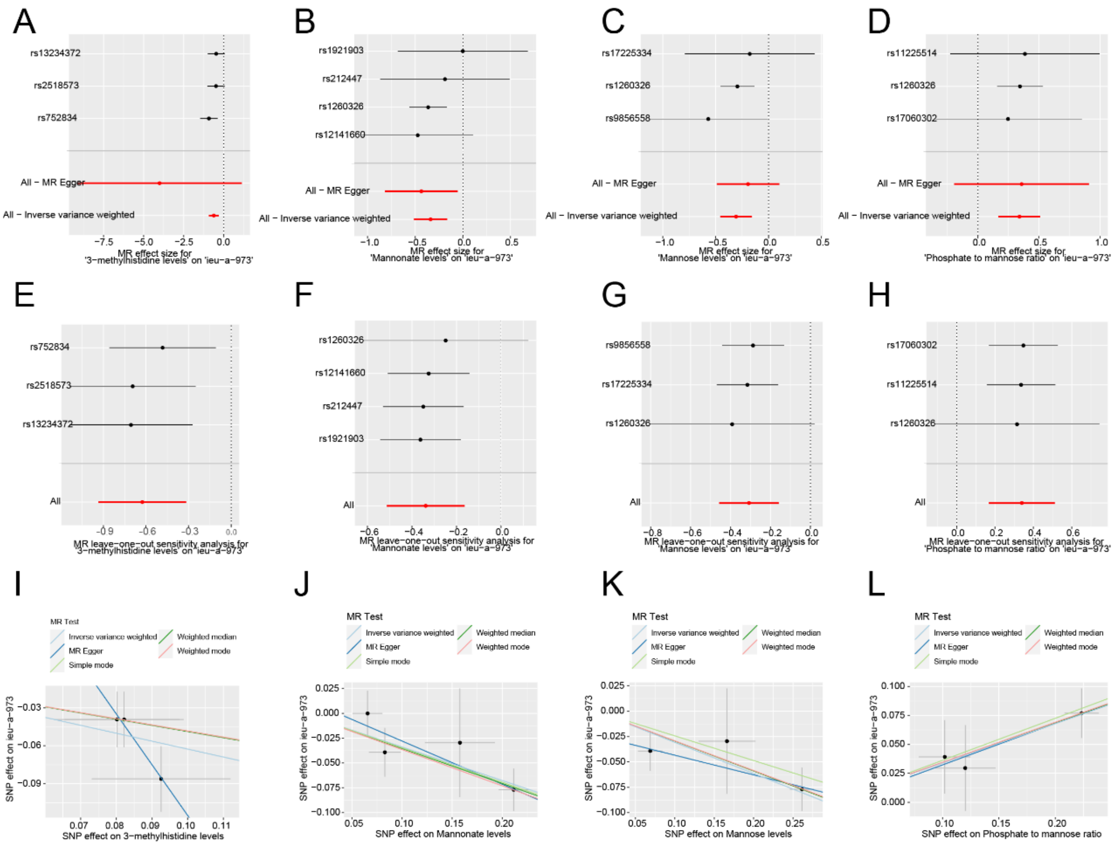


**Figure S2. A-D**, Forest plot of the causal relationship between metabolites and UC risk. **E-H**, leave-one-out plot to visualize causal effect of metabolites on total UC risk when leaving one SNP out. **I-L**, Scatter plots of Mendelian randomization analysis.

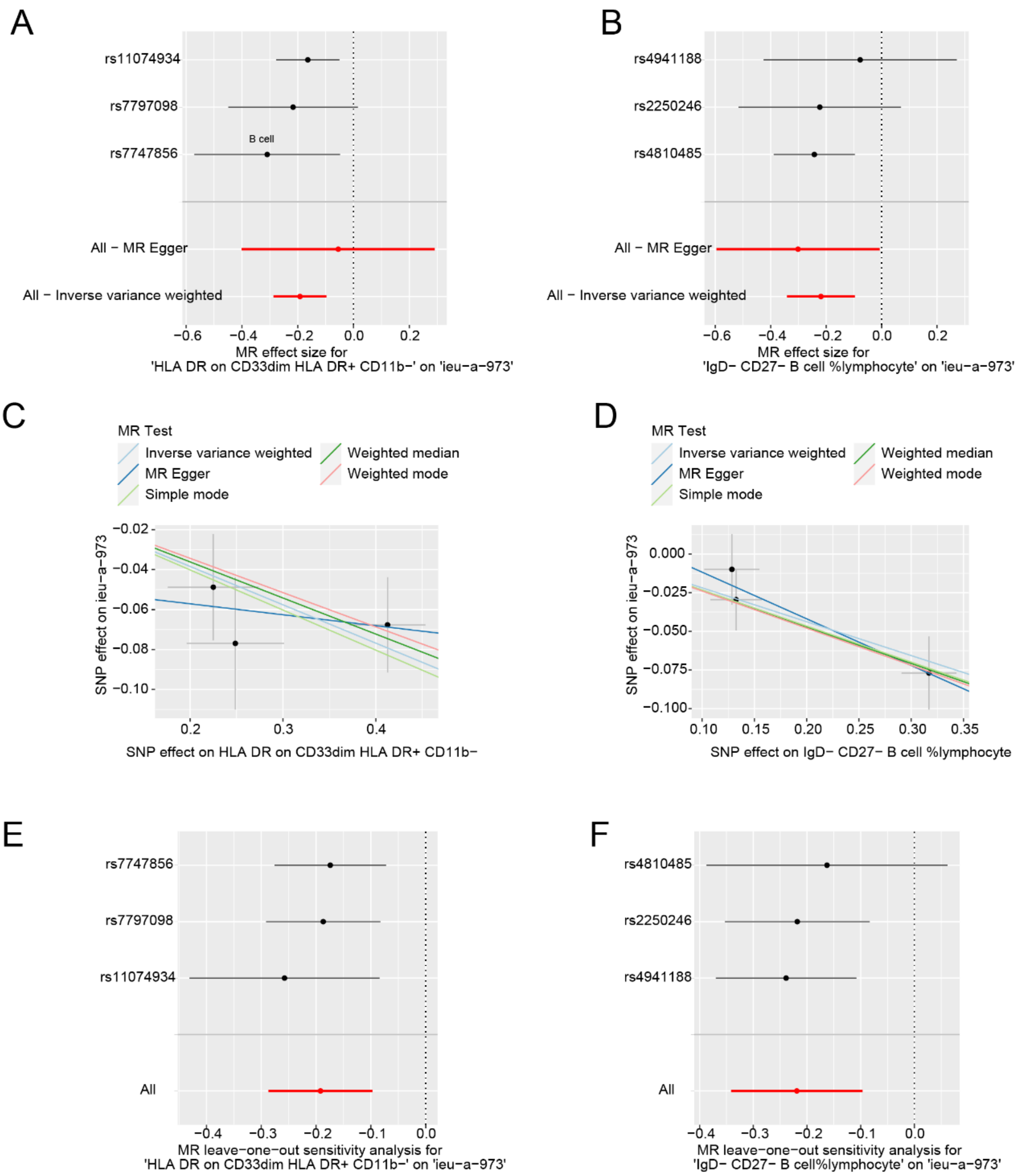


**Figure S3. A-B**, Forest plot of the causal relationship between immune cell phenotypes and UC risk. **C-D**, Scatter plots of Mendelian randomization analysis. **E-F**, leave-one-out plot to visualize causal effect of immune cell phenotypes on UC risk when leaving one SNP out.

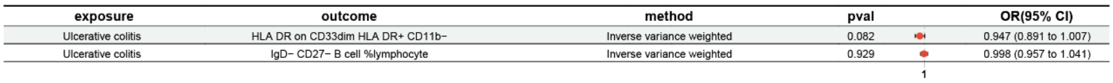

| exposure | outcome | method | pval | | OR(95% CI) |
|---|---|---|---|---|---|
| Ulcerative colitis | HLA DR on CD33dim HLA DR+ CD11b− | Inverse variance weighted | 0.082 | | 0.947 (0.891 to 1.007) |
| Ulcerative colitis | IgD− CD27− B cell %lymphocyte | Inverse variance weighted | 0.929 | | 0.998 (0.957 to 1.041) |

**Figure S4.** Forest plot showing the reverse Mendelian randomization analysis of the causal relationship between immune cell phenotypes and UC risk

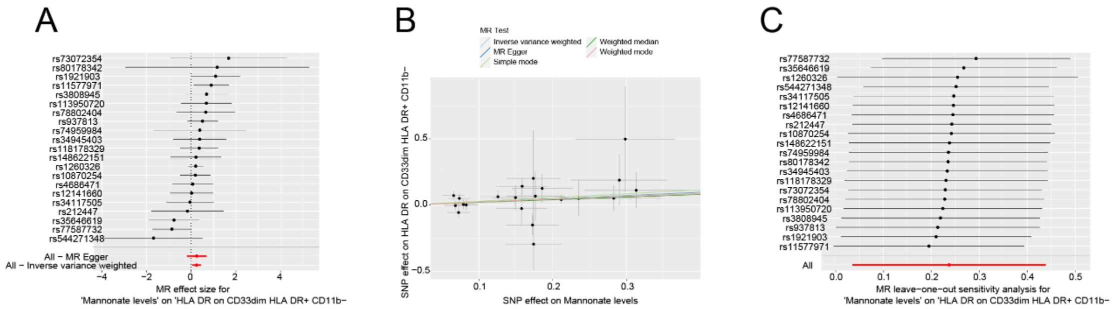


**Figure S5.** **A**, Forest plot of the causal relationship between metabolites and immune cell phenotypes. **B**, Scatter plots of Mendelian randomization analysis. **C**, leave-one-out plot to visualize causal effect of metabolites on immune cell phenotypes when leaving one SNP out.

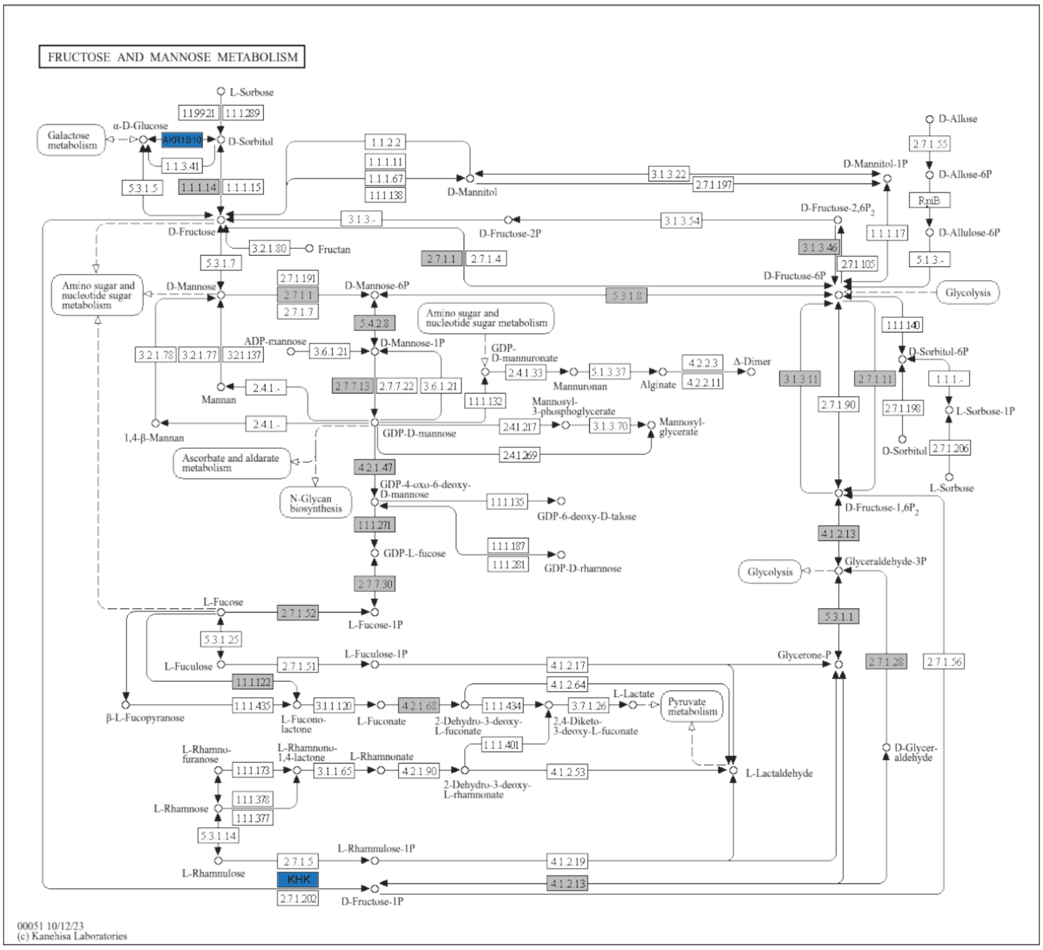


**Figure S6.** The role of KHK and AKR1B10 in the fructose and mannose metabolism pathway.
