# Supplementary material for: Multiple Comprehensive Analyses Identify the Protective Role and Diagnostic Signature of Mannose Metabolism in Ulcerative Colitis
Source: Int J Mol Sci. 2025 Sep 26;26(19):9443. doi: 10.3390/ijms26199443 (PMC12524464; doi:10.3390/ijms26199443)
Supplement: Supplementary file 1 [file ijms-26-09443-s001.zip › Supplementary File S1.pdf]

Primers for qRT-PCR

Transcript	Forward	Reverse
**IL-6**	ACTCACCTCTTCAGAACGAATTG	CCATCTTTGGAAGGTTCAGGTTG
**IL-1β**	CAGTGGCAATGAGGATGACTTGTTC	CTGTAGTGGTGGTCGGAGATTCG
**KHK**	CAGCGGATAGACGCACACAA	GCCACATCTTTGCTGACAAAC
**AKR1B10**	GTGACACCAGCACGCATTG	GCATTGAAGGGATAGTCTTCCAA
**GAPDH**	CAGGAGGCATTGCTGATGAT	GAAGGCTGGGGCTCATTT
